# Effect of Ultraviolet-B Radiating *Drosophila melanogaster* as Host on the Quality of *Trichopria drosophilae*, a Pupal Parasitoid of *Drosophila suzukii*

**DOI:** 10.3390/insects14050423

**Published:** 2023-04-28

**Authors:** Xuxiang Liu, Yongbang Yang, Qingwen Fan, Qinyuan Zhang, Qinge Ji

**Affiliations:** 1 Biological Control Research Institute, Plant Protection College, Fujian Agriculture and Forestry University, Fuzhou 350002, China; liuxx1003@163.com (X.L.); yyb1234560904@163.com (Y.Y.); 18336086200@163.com (Q.F.); congcong9758@163.com (Q.Z.); 2 China Fruit Fly Research and Control Center of FAO/IAEA, Fuzhou 350002, China; 3 Key Lab of Biopesticide and Chemical Biology, Ministry of Education, Fuzhou 350002, China; 4 State Key Laboratory of Ecological Pest Control for Fujian and Taiwan Crops, Fuzhou 350002, China

**Keywords:** biological control, natural enemy, mass rearing, ultraviolet radiation, increasing efficiency

## Abstract

**Simple Summary:**

The mass rearing of parasitoids is a basic prerequisite for their use in biological control. If parasitoid rearing is effective, host/parasitoid mixtures will not form. In this study, we used an irradiated *Drosophila melanogaster* host to supply parasitoids and assessed whether all adults that emerged after parasitization were *Trichopria drosophilae*. The results of this study showed that irradiation of host pupae for 6 h is the preferred treatment for promoting the emergence and therefore the potential application of *T. drosophilae* in the control of *Drosophila* pests. At the same time, the results also serve as an important reference for reducing costs and improving the efficiency of biological pest control.

**Abstract:**

The pupal parasitoid, *Trichopria drosophilae* Perkins (Hymenoptera: Diapriidae), is an ectoparasitoid of the genus *Drosophila* with great potential for application in biological control based on its excellent control efficiency for *Drosophila suzukii* Matsumura (Diptera: Drosophilidae), and it has has even been commercialized by biofactories. Due to its characteristics of short life cycle, large number of offspring, easy rearing, rapid reproduction, and low cost, *Drosophila melanogaster* (Diptera: Drosophilidae) is currently being utilized as a host to mass produce *T. drosophilae*. To simplify the mass rearing process and omit the separation of hosts and parasitoids, ultraviolet-B (UVB) was used as an irradiation source to irradiate *D. melanogaster* pupae, and the effects on *T. drosophilae* were studied. The results showed that UVB radiation significantly reduces host emergence and affects the duration of parasitoid development (female: F0 increased from 21.50 to 25.80, F1 from 23.10 to 26.10; male: F0 decreased from 17.00 to 14.10, F1 from 17.20 to 14.70), which has great significance for the separation of hosts and parasitoids as well as of females and males. Of the various studied conditions, UVB irradiation was ideal when the host was supplied with parasitoids for 6 h. The selection test results showed that the female-to-male ratio of emerging parasitoids in this treatment was highest at 3.47. The no-selection test resulted in the highest rates of parasitization and parasitoid emergence rate, maximized inhibition of host development, and allowed the omission of the separation step. Finally, the results of the semi-field test showed that the parasitoids bred in this treatment could search for their hosts normally and could therefore be directly applied in the biological control of *Drosophila* pests in the field.

## 1. Introduction

*Drosophila melanogaster* Meigen (Diptera: Drosophilidae) is well known as one of the most intensively studied model insects [1,2]. In contrast to this saprophageous species, *Drosophila suzukii* Matsumura (Diptera: Drosophilidae) feed on fresh fruits. [3,4]. *Drosophila suzukii* females have serrated ovipositors and lay eggs in the sarcocarp, where the larvae hatch and feed inside the fruit, greatly accelerating the rate of decay and deterioration. At the same time, the physical damage caused to the fruit by eggs of *D. suzukii* females can lead to secondary infestation by other organisms [5,6]. This species is native to Asia, and it has invaded Europe, North and South America, Africa, and Oceania [7]. It has quite high ecological adaptability, wide host range, short generation cycle, and causes serious harm and significant economic losses [4,8,9]. At present, the methods of controlling *D. suzukii* are mostly physical (especially in cherry orchards), chemical (insecticides), and biological (bacteria, viruses, fungi, nematodes, parasitoids, and predators), with other forms, too [10]. The main focus is on chemical control, but this method tends to cause 3R (Resistance, Residue, Resurgence) problems and does not adequately address the root cause of the damage [11,12]. The biological control of *D. suzukii* is already being applied, although there is certainly room for increased application. In this context, the biological control of *D. suzukii* with parasitoids shows excellent application prospects [13,14].

*Trichopria drosophilae* Perkins (Hymenoptera: Diapriidae), an important endoparasitoid of *Drosophila*, can not only successfully parasitize *D. suzukii*, but can also produce offspring that can smoothly emerge into parasitoids, making them a perfect candidate as a biological control parasitoid against *D. suzukii* [15,16,17,18,19]. *Trichopria drosophilae* is currently distributed in China [17,18], North America [17,20,21], France [22,23], Korea [24], Switzerland [25], Mexico [17,20,21], Italy [26], Spain [14], and other countries, and has been reported from Shandong [27], Zhejiang [28], Fujian [18], Yunnan [29], and Anhui [30] in China. *Trichopria drosophilae* prefers to parasitize fly pupae that are hidden inside fruit rather than those buried in the soil. Parasitoids can easily and efficiently search for both types of pupae in different locations, and when they find their hosts, can paralyze them and lay eggs directly inside [17,19]. The endoparasitoid, *T. drosophilae*, has significantly higher efficiency than the *Drosophila* ectoparasitoid, represented by *Pachycrepoideus vindemmiae* Rondani (Hymenoptera: Pteromalidae) [18,31], and *T. drosophilae* is also a suppressive solitary parasitoid that kills its hosts while parasitizing them [14,22]. When parasitizing alone, *T. drosophilae* is significantly more efficient than *P. vindemmiae*. When parasitizing together, *T. drosophilae* is more likely to affect *P. vindemmiae*. *Trichopria drosophilae* can parasitize a relatively small number of species compared with *P. vindemmiae*, which can parasitize more than 60 species of flies [13,26]. However, *T. drosophilae* can successfully parasitize *D. suzukii*. It has been shown that two larval parasitoids of Figitidae, *Leptopilina heterotoma* Thomson, and *L. boulardi* Barbotin can also parasitize *D. suzukii* [32,33]. However, neither parasitoid is able to complete development on *D. suzukii* because diptera larvae develop a relatively strong immune response to them, with a high encystment rate of 74% for *L. heterotoma* eggs and a relatively low encystment rate of 52% for *L. boulardi* eggs, which is an important reason for the failure of both to complete development [22]. Although the immune response has led to poor control of flies by larval parasitoids, the pupal parasitoids represented by *T. drosophilae*, have shown great adaptation to this immune response. *T. drosophilae* is thus a highly promising resource for the biological control of fly pests as a natural enemy of parasitoids [34,35].

To achieve the application of parasitoids for the control of *D. suzukii*, large numbers of host pupae are required. At the same time, its pupae are difficult to obtain, and *D. melanogaster* has potential as a model insect for use as an alternative to *D. suzukii* parasitoids as a breeding host because of its short life cycle, large number of offspring, easy sex differentiation and rearing, fast reproduction, and low cost [1,2]. However, the rearing process requires that parasitoids be separated from their unparasitized hosts, which may be addressed through the use of ultraviolet radiation. The intensity of ultraviolet radiation varies widely in space, and radiation gradients can control the movement of organisms and their performance [36]. Ultraviolet radiation inhibits the incidence of fly ovipositing; reduces the survival rate of eggs, the life span of adults, and the duration of development; and has detrimental effects on the reproductive organs of offspring, leading to a certain degree of teratogenicity in offspring while affecting their phenotype and behaviour [35,36,37,38]. At the same time, ultraviolet radiation enhances the parasitic effect of parasitoids on their hosts in many ways. Ultraviolet radiation affects the selection and suitability of hosts for parasitoids, which in turn impacts the effectiveness of parasitoids [39,40,41,42,43,44,45]. The parasitization of *Trichogramma chilonis* Ishii (Hymenoptera: Trichogrammatidae) was shown to be significantly increased in ultraviolet-irradiated hosts compared to unirradiated host eggs. There was also a significant increase in *T. chilonis* emergence from hosts, and radiation inhibited the development of unparasitized hosts. In addition, compared with gamma irradiation, the host treated with ultraviolet radiation is more likely to be parasitized, and ultraviolet radiation treatment is more suitable for rearing and extension applications of biological natural enemies [42,46]. If ultraviolet irradiation can achieve lower levels of host emergence, in addition to its features of host suitability and parasitoid efficiency, this would result in greatly improved rearing efficiency.

This study aims to determine the optimal host irradiation dose to achieve the highest parasitism rate in simulated outdoor field environment conditions. The offspring parasitoids are obtained using irradiated pupae as hosts, and the parasitized environment under natural conditions is restored to the maximum extent, so as to evaluate their effectiveness. After a series of experiments on the effects of ultraviolet radiation on hosts, UVB was selected as a suitable test radiation for irradiating hosts [35]. In this study, *D. melanogaster* pupae irradiated with different doses of UVB were used as hosts to rear *T. drosophilae* with assessment of the parasitism rate, emergence rate, sex ratio, pupal death rate, development duration, longevity, and outdoor parasitization performance of *T. drosophilae*. This research will provide compelling support for the mass rearing of natural enemy resources and simplify the rearing process. It will also provide basic information on the effects of the use of ultraviolet-irradiated hosts on the development of parasitoids.

## 2. Materials and Methods

### 2.1. Insects

Laboratory colonies of *D. melanogaster* and *T. drosophilae* were collected from ecological orchards (119°13′39.98″ E, 25°42′36.00″ N) in Fuqing City, Fuzhou City, Fujian Province, P.R. China. The trap box was a square plastic box with an escape-prevention device and the exterior of the box was wrapped with alternating black, red, and yellow tape to attract flies [47,48,49,50]. The box contained fruit that had just started to rot and an artificial diet for flies. At the same time, *T. drosophilae* were trapped using a box containing a skimmed cotton ball soaked with honey and pupae of *D. suzukii* and *D. melanogaster*.

The collected insects were reared indoors and maintained under controlled conditions (25 ± 1 °C, 65 ± 5% RH, 12:12 h (L/D)). In order to ensure fresh food and clean water and to avoid mildew adversely affecting insect sources, the food and water were kept adequately well supplied and replaced regularly. The gauze cage was covered on all six sides with 100-mesh nylon that had a cylindrical operating cuff (L: 30.00 cm, D: 10.00 cm) on one side to facilitate handling inside the cage. Adult *D. melanogaster* were maintained in cages and supplemented with a standard cornmeal-based artificial diet. The artificial diet formula contained cornmeal: 50.00 g, sucrose: 40.00 g, brewery yeast: 20.00 g, agar: 5.00 g, 36% *v*/*v* acetic acid: 3.00 mL, 95% *v*/*v* ethanol: 7.00 mL, ethyl p-hydroxybenzoate: 1.00 g, and sodium benzoate: 1.00 g [17,18,19]. The pupae of *D. melanogaster* were placed in a *T. drosophilae* cage for 24 h and then placed in a new cage until they emerged. The nutriment of *T. drosophilae* adult was fresh 10% honey water.

### 2.2. UVB Radiation

UVB lights (Nanjing Huaqiang Electronics Co., Ltd., Nanjing, China), which could emit 300 nm UVB, were used as the source to irradiate newly collected pupae for different durations at 50.00 cm above the pupae.

### 2.3. Host Species Preference

A selection test was designed to evaluate whether *T. drosophilae* preferred to parasitize treated *D. melanogaster* pupae. Firstly, newly formed *D. melanogaster* pupae (within 24 h) were taken, and the collected pupae were placed in Petri dishes lined with moistened filter paper and irradiated to UVB for 0, 3, 6, and 9 h. From each test group corresponding to 4 treatments, 25 pupae were taken, with 100 pupae taken in total. The pupae were placed on a designated quarter of a moistened filter paper in a Petri dish (d: 9.00 cm, h: 2.00 cm) using the cross-division method and provided with 10 pairs (female/male = 1:1) of sexually mature virgin *T. drosophilae* for 24 h. Each treatment included five replicates. After parasitization, the pupae of the four treatments were separated. The number and sex of parasitoids that emerged in each treatment were counted separately and used to calculate the number of emerged parasitoids and the female-to-male sex ratio.

### 2.4. Parasitic Efficiency Based on Generations

A no-selection test aimed to evaluate the parasitic ability of *T. drosophilae* on differently treated fly pupae. First, newly formed *D. melanogaster* pupae (within 24 h) were taken, and the collected pupae were placed in Petri dishes lined with moistened filter paper and irradiated to UVB for 3, 5, 6, 7, and 9 h. An additional negative control was set up without radiation. Irradiated flies pupae were placed individually in Petri dishes lined with moist filter paper and provided with 10 pairs (female/male = 1:1) of sexually mature virgin *T. drosophilae* for 24 h. One hundred pupae were taken from each treatment and each treatment included three replicates. This generation treatment process was recorded as F0.

To wait for the emergence, 100 treated pupae were placed in a Petri dish. The number and sex of parasitoids were recorded daily and used to calculate the emergence rate. Pupae from which an adult (either parasitoid of fly) did not emerge were dissected to identify whether the pupae contained parasitoids or flies. The sex of parasitoids was considered based on the type of antennae or the presence of an ovipositor on the abdomen, and the amount corresponding to each sex was counted. The above data were used to calculate the parasitism rate and sex ratio (female/male). Pupae that did not emerge and died of blackening were recorded as the number of dead pupae and were used to calculate the pupal death rate. The development duration of parasitoids was from the completion of treatment to the emergence of parasitoids. At the same time, newly emerging *T. drosophilae* were randomly selected from each treatment and provided with honey and water but no host under controlled conditions (25 ± 1 °C, 65 ± 5% RH, 12:12 h (L/D)). The survival conditions of *T. drosophilae* were recorded every day to calculate their lifespan and the dead *T. drosophilae* were cleaned up at the same time. Ten adults of each sex were used in each replicate of the development duration and longevity test. A certain number of newly emerged male and female parasitoids were randomly selected from each treatment, and the above-described no-selection experiments were repeated, with the same statistical indicators and computational methods as above. This generation treatment process is recorded as F1.

### 2.5. Outdoor Test

The newly formed pupae of *D. melanogaster* (within 24 h) were treated with UVB for 6 h and then served as hosts. The proportion of pupae to female parasitoids was 1:10, and sexually mature virgin *T. drosophilae* were provided for 12 h of outdoor parasitization. A negative control without radiation was also set up, and each treatment included three replicates. After parasitization, the pupae were placed in the parasitoid rearing room for the emergence of parasitoids, recorded as F1. The parasitoids were separately packed and supplemented with honey water and kept until the parasitoids were sexually mature. After normal pupae were provided and parasitized in accordance with the above proportion, the emerging parasitoids were recorded as F2.

The outdoor test was carried out in a bioassay cage (30.00 cm × 30.00 cm × 30.00 cm, covered on all six sides with 100 mesh nylon) with the following conditions: a surface lined with 100 pupae of halved cut grapes for parasitoids and cotton moistened with honey water as a nutritional supplement for the parasitoids. Twenty sexually mature virgin parasitoids (female/male = 1:1) were released into cages, grapes were collected after 12 h, and the pupae were brushed onto Petri dishes lined with moistened filter paper, and emergence was awaited. Both generations were tested. The measures of F1 and F2 parasitoids quality for outdoor testing and calculations were the same as the no-selection test in Section 2.4.

### 2.6. Data Analysis

WPS Office 2022 (Kingsoft Co., Ltd., Beijing, China) was used to count raw data and calculate parasitism rate, emergence rate, sex ratio, pupal death rate, development duration, and longevity, and also for plotting. All data were analyzed using one-way ANOVA with SPSS v.23.0 (SPSS Inc., Chicago, IL, USA), and multiple comparisons were performed using the LSD method. Statistical results were expressed as mean ± SD, with *p* < 0.05 considered to indicate statistical significance, and multiple comparison results were marked according to the letter-marking method.

## 3. Results

### 3.1. Host Species Preference

**Figure 1:**
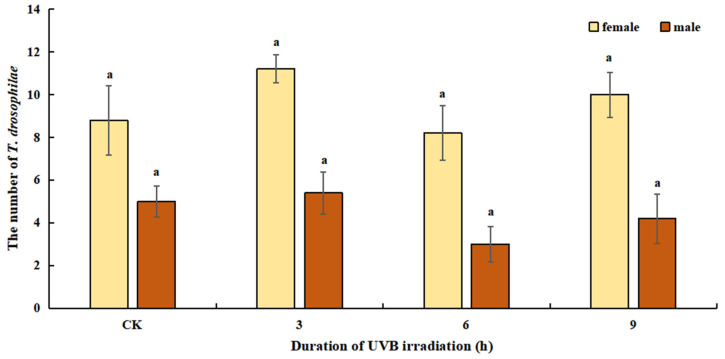
Effect of UVB-irradiated pupae on emergence amount of *Trichopria drosophilae* (number). Here and below, bars refer to mean ± SE and different lowercase letters above the bars indicate significant differences (Duncan’s LSD, *p* < 0.05). CK means control with no irradiated treatment.

In the selection test, there was no significant difference in the number of females (*F*_3, 16_ = 1.216, *p* = 0.336) and males (*F*_3, 16_ = 1.280, *p* = 0.315) that emerged between all treated hosts. The test results show that parasitoids preferred the host pupae of B3 (UVB and irradiation hours; similar below), from which 16.80 parasitoids emerged, corresponding to an emergence rate of 67.20%. The numbers of females and males that emerged were 11.20 and 5.40, respectively, which was not significantly different from the control (*p* = 0.179, *p* = 0.766). Meanwhile, 14.20 parasitoids emerged from *T. drosophilae* parasitized hosts of B9, an emergence rate of 56.80%. Of these, there were 10.00 females and 4.20 males, both of which were not significantly different from the control (*p* = 0.492, *p* = 0.554, Figure 1).

**Figure 2:**
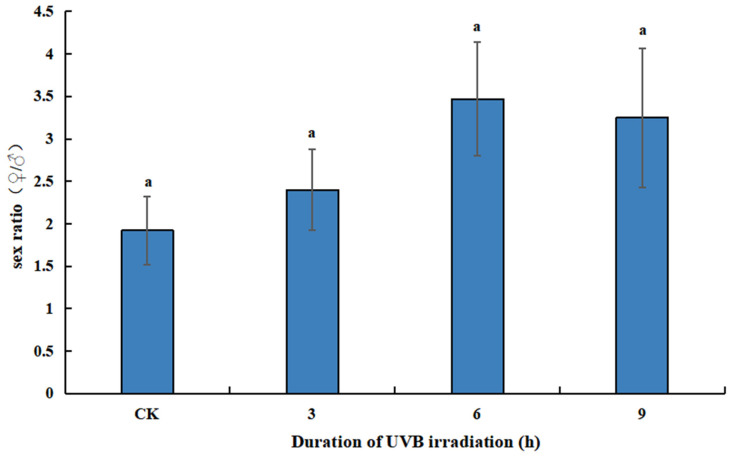
Effect of UVB-irradiated pupae on the sex ratio of *T. drosophilae*.

In the selective host test, there was no significant difference in sex ratios of the parasitoids that emerged from differently treated hosts (*F*_3, 16_ = 1.396, *p* = 0.280). The sex ratios of parasitoids that emerged from B6, B9, and B3 were 3.47, 3.25, and 2.40, respectively, all of which were higher than but not significantly different from the control (*p* = 0.150, *p* = 0.554, *p* = 0.766, Figure 2).

### 3.2. Parasitic Efficiency Based on Generations

#### 3.2.1. Effect of UVB-Irradiated Pupae on Parasitism Rate, Emergence Rate, Sex Ratio, and Pupal Death Rate of *T. drosophilae*

**Figure 3:**
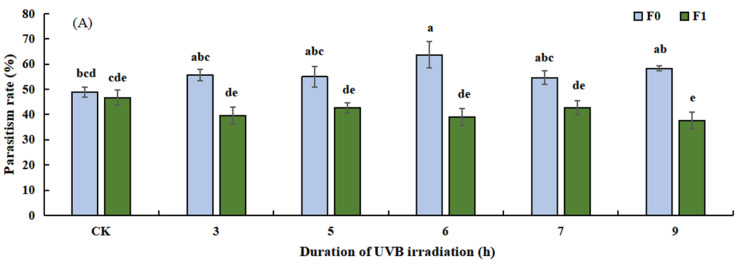

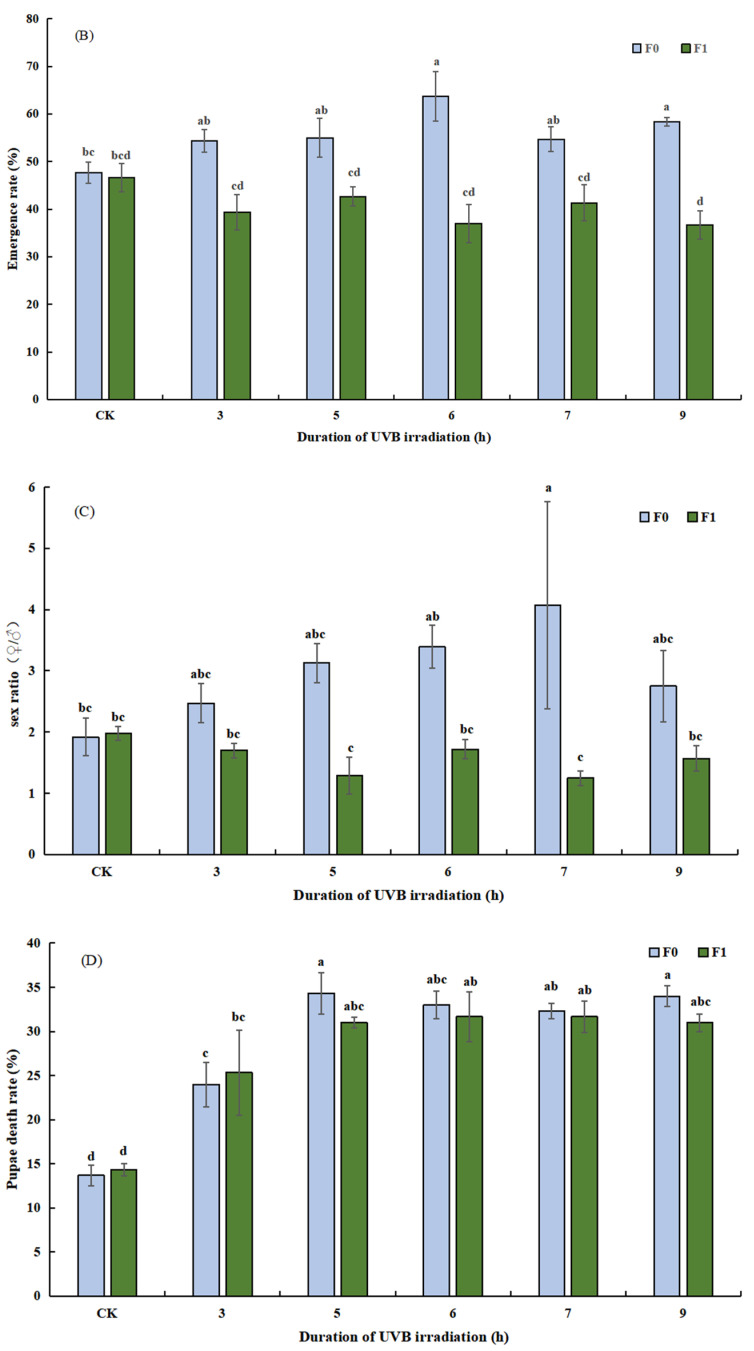
Effects of UVB-irradiated pupae on parasitism rate (**A**), emergence rate (**B**), sex ratio (**C**) and pupal death rate (**D**) of *Trichopria drosophilae*.

Ultraviolet radiation significantly affected the parasitism rate (*F*_11, 24_ = 7.643, *p* = 0.000, Figure 3A), emergence rate (*F*_11, 24_ = 7.555, *p* = 0.000, Figure 3B), sex ratio (*F*_11, 24_ = 2.541, *p* = 0.027, Figure 3C), and pupal death rate (*F*_11, 24_ = 10.380, *p* = 0.000, Figure 3D) of *T. drosophilae*. There were significant differences in the parasitism rate (*p* = 0.001, *p* = 0.010, *p* = 0.000, *p* = 0.012, *p* = 0.000, Figure 3A) and the emergence rate (*p* = 0.003, *p* = 0.013, *p* = 0.000, *p* = 0.008, *p* = 0.000, Figure 3B) between F0 and F1. For B5, B6, and B7, the F0-to-F1 sex ratio (*p* = 0.030, *p* = 0.046, *p* = 0.002, Figure 3C) was significantly different. This somewhat corroborated the need for two more treatments (B5 and B7) in the no-selection compared with the selection test.

#### 3.2.2. Effect of UVB-Irradiated Pupae on the Duration of Development and Longevity of *T. drosophilae*

**Figure 4:**
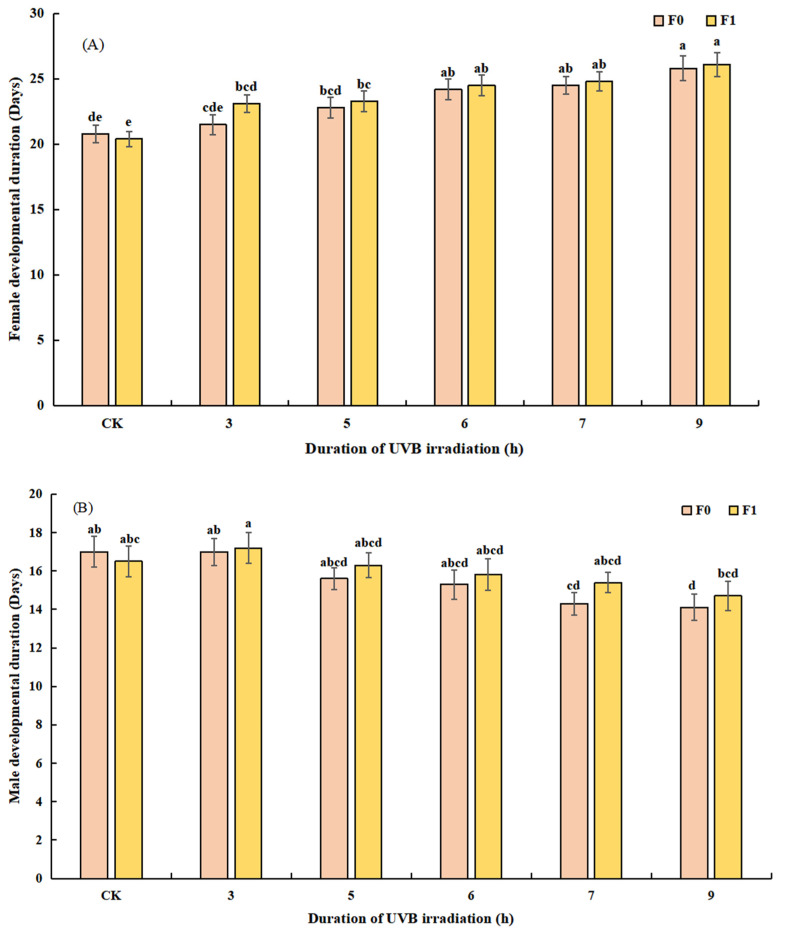
Effects of UVB-irradiated pupae on female (**A**) and male (**B**) development duration of *Trichopria drosophilae*.

UVB radiation significantly affected the duration of development of female (*F*_11, 108_ = 5.840, *p* = 0.000, Figure 4A) and male (*F*_11, 108_ = 2.225, *p* = 0.018, Figure 4B) *T. drosophilae*. The duration of development for females increased with rising radiation time, with F0 increasing from 21.50 ± 0.75 (range: 19–27 d) in B3 to 25.80 ± 0.95 (range: 22–31 d) in B9. F1 increased from 23.10 ± 0.69 (range: 20–26 d) in B3 to 26.10 ± 0.91 (range: 22–30 d) in B9. In contrast to the trend shown by females, the duration of development for males decreased with rising radiation time. F0 shortened from 17.00 ± 0.70 (range: 14–21 d) in B3 to 14.10 ± 0.69 (range: 11–19 d) in B9. F1 shortened from 17.20 ± 0.81 (range: 14–21 d) in B3 to 14.70 ± 0.76 (range: 11–19 d) in B9.

**Figure 5:**
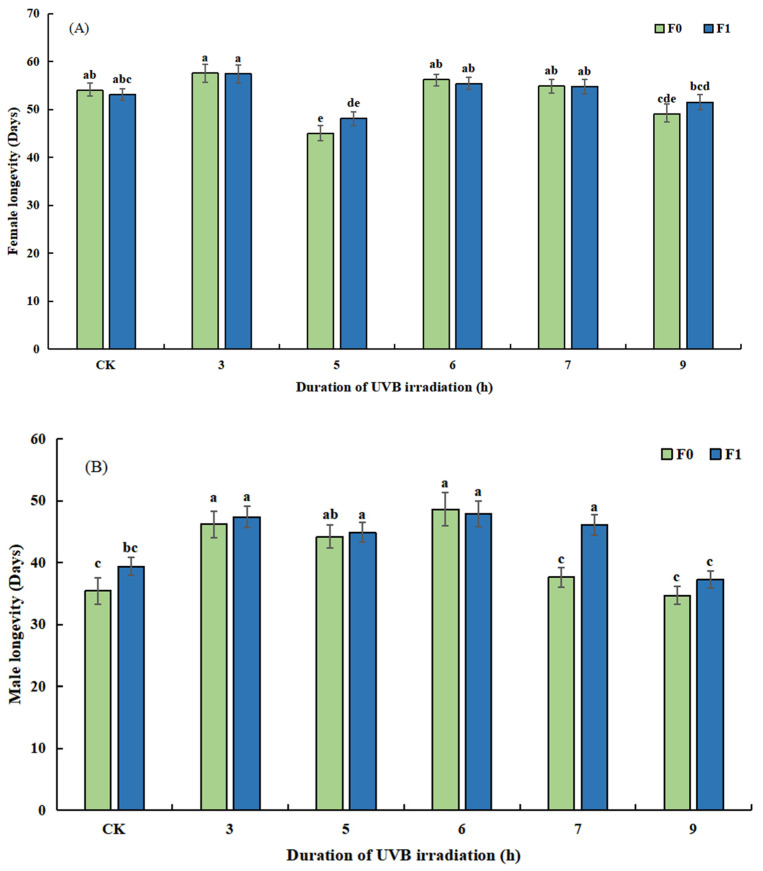
Effects of UVB-irradiated pupae on female (**A**) and male (**B**) longevity of *Trichopria drosophilae*.

UVB radiation significantly affected the longevity of female (*F*_11, 348_ = 6.280, *p* = 0.000, Figure 5A) and male (*F*_11, 348_ = 7.875, *p* = 0.000, Figure 5B) *T. drosophilae*. In both F0 and F1 generations, the shortest female longevity was in B5, 45.03 ± 1.57 (range: 28–57 d) and 48.13 ± 1.45 (range: 31–58 d), respectively, which was significantly different from control (*p* = 0.000, *p* = 0.025). B3 had the longest female longevity of both F0 and F1, 57.60 ± 1.83 (range: 37–73 d) and 57.47 ± 1.86 (range: 37–73 d), respectively, which was not significantly different from control (*p* = 0.102, *p* = 0.052). For male longevity, the shortest male longevity was 34.70 ± 1.48 (range: 17–54 d) and 37.27 ± 1.41 (range: 22–55 d) in B9 for both F0 and F1 generations. B6 had the longest male longevity of both F0 and F1, 48.63 ± 2.68 (range: 31–78 d), and 47.87 ± 2.11 (range: 32–68 d), respectively.

### 3.3. Determination of the Outdoor Quality of Reared Irradiated Pupae Based on F1 and F2 T. drosophilae

#### 3.3.1. Outdoor Quality of Reared UVB-Irradiated Pupae Based on Parasitism Rate, Emergence Rate, Sex Ratio, and Pupal Death Rate of F1 and F2 *T. drosophilae*

Radiation significantly affected the parasitism rate (*F*_2, 6_ = 25.289, *p* = 0.001), emergence rate (*F*_2, 6_ = 32.250, *p* = 0.001), sex ratio (*F*_2, 6_ = 10.981, *p* = 0.010), and pupal death rate (*F*_2, 6_ = 53.356, *p* = 0.000) of F1 *T. drosophilae* (Table 1). The measured numerical values of parasitism rate, emergence rate, sex ratio, and pupal death rate were lower outdoors than indoors with the same treatment, but all four indexes were higher and significantly different from the control after radiation (*p* < 0.001, *p* < 0.001, *p* = 0.003, *p* < 0.001). In comparison to F1 and F2, all indexes were lower in F2 than in F1, but still higher than the control. Overall, the parasitoids produced from irradiated pupae could normally search for hosts outdoors and emerge into adult parasitoids.

#### 3.3.2. Outdoor Quality of Reared UVB-Irradiated Pupae Based on Duration of Development and Longevity of F1 and F2 *T. drosophilae*

A comparison of outdoor quality between F1 and F2 *T. drosophilae* from irradiated and contrasted pupae is shown in Table 2, including the duration of development (females: *F*_2, 27_ = 3.766, *p* = 0.036; males: *F*_2, 27_ = 0.792, *p* = 0.463) and longevity (females: *F*_2, 87_ = 0.894, *p* = 0.413; males: *F*_2, 87_ = 1.873, *p* = 0.160) of parasitoids. For the F1 generation, the female duration of development was significantly different from the control (*p* = 0.047). The differences in the two parameters between F1 and F2 generations of both males and females were less pronounced, and only the duration of development of females differed significantly between F1 (range: 20–29 d) and F2 (range: 17–26 d) (*p* = 0.015). There were no significant differences between F1 and F2 generations in the duration of development of males (F1 range: 13–20 d, F2 range: 13–22 d) and the longevity of both males (F1 range: 30–63 d, F2 range: 20–60 d) and females (F1 range: 41–67 d, F2 range: 30–63 d) (*p* = 0.255, *p* = 0.389, *p* = 0.262, Table 2).

## 4. Discussion

A number of studies on the effects of radiation on organisms have been applied to insects because of their easy handling, short life cycle, large number of offspring produced by most species, and other favorable biological characteristics [51]. These processes include traditional gamma rays, emerging ultraviolet rays, and microwaves [52,53,54,55]. Ultraviolet radiation affects the adaptation of eggs, larvae, pupae, and adult stages of insects, encompassing all periods of insect development and showing great potential for application in integrated pest management [42,56]. The ultraviolet part of the spectrum is widely used for sterilization, surface disinfection of insect eggs, insect attraction and extermination, and inhibition of different stages of the insect life cycle [57,58,59,60]. Insects may be at high risk from ultraviolet radiation due to their small size. Radiation may penetrate much more deeply in insect tissues than larger organisms, thus disproportionately affecting their performance [36].

*Trichopria drosophilae* is widely used in the biological control of flies, and it shows great flexibility in host selection [26,28,61,62]. Host species, age, and treatment methods can significantly affect parasitization of the parasitoids [63,64]. Our study is the first to both assess the suitability of *T. drosophilae* for UVB radiation of hosts and carry out quality test evaluation via an outdoor test. The results show that UVB radiation can affect the parasitic efficiency and performance of *T. drosophilae*, which can influence the suitability of hosts for parasitoids. Similar studies have shown that *Trichogramma chilonis* prefers to parasitize ultraviolet-irradiated host eggs compared to fertilized and unfertilized eggs. Therefore, irradiation improves the adaptability of parasitoids to hosts [43]. Furthermore, the emergence rate of 1-day-old pupae of *Plutella xylostella* Linnaeus (Lepidoptera: Plutellidae) was significantly decreased by UVB irradiation [65]. This effect may be caused by the changes in the host immune system under the influence of ultraviolet radiation during the co-evolution of the hosts and parasitoids [66]. Ultraviolet radiation can kill hosts in a certain stage and retain host nutrients for the growth and development of parasitoids, which may partially explain why parasitoids prefer to parasitize hosts after ultraviolet radiation [43]. Our selection test showed that more parasitoids emerged from hosts treated with short UVB, but the difference was not significant with others. Combining the result of the ratio of females to males in the selection test with previous studies on irradiated hosts, we can infer that B6 should be a more suitable means of treating hosts [35]. The host treated with UVB was parasitized by *T. drosophilae*, and the parasitism rate was higher than the emergence rate. A small proportion of parasitized hosts did not successfully emerge from *T. drosophilae*, probably because the short radiation of hosts had an effect on the growth and development of parasitoids within the host. Some *T. drosophilae* failed to become normal individuals, or their development was impaired, or they were even deformed, thus preventing some of them from successfully emerging. If the test host is *D. suzukii,* with a strong immune response, it is worth investigating whether this loss can be reduced to a certain extent in future experiments. Doing so will pave the way for the successful application of this technique to more species of *Drosophila* pest control. The use of microscopy in this study revealed that a significantly lower number of *T. drosophilae* in the treatment group were incompletely developed, but this was even lower in the control. The increased rate of dead pupae after ultraviolet radiation also indicates that the growth and development of the host and *T. drosophilae* are affected by treatment to a certain extent. Meanwhile, radiation increased the parasitic efficiency of parasitoids on their hosts. Unfortunately, after two generations of testing, this increase in efficiency was not passed to the next generation. A scientific question worth investigating is whether successive generations of irradiated treated hosts supplying parasitoids with parasitization will allow this tendency to be passed to future generations. With the increase in irradiation time from 3 h to 5 h in the no-selection test, the mortality of host pupae gradually increased. However, this trend almost completely disappeared later as the treatment time continued to increase. A follow-up test should be conducted to explore whether the pupae have a certain tolerance or upper limit to irradiation. The longevity of male and female parasitoids of B5 was lower than B3 and B6. Whether this treatment duration could be used as a special method to affect the survival of parasitoids needs to be further tested to verify its feasibility.

The connections between insects and microbes are wide-ranging and influential [67]. Among the adaptive effects of microbes on hosts, defense against natural enemies is increasingly considered to be universal, especially in those associations involving heritable and facultative bacteria [68]. The study of the interaction between *Drosophila*, bacteria (Spiroplasma MSRO), and parasitoids has laid the foundation for our understanding of the important roles played by each part of symbionts. Moreover, MSRO is also a male killer. In this study, the developmental trends of female and male parasitoids were in stark contrast. Whether treatment affected the survival of microbes and their interactions and then affected the different adaptability of parasitoids of different sexes to treatment is a question to be scientifically explored [69]. Maternally inherited bacteria, meanwhile, are common in many insects but are generally unculturable, maintaining their interests by manipulating their host to reproduce or giving it an adaptive advantage [68]. This benefit may depend on the environment to resist abiotic stress or natural enemies. It remains to be explored whether the radiation treatment in this study will affect their survival and further prolong the development duration of female parasitoids. After UVB radiation, males and females showed different developmental trends, indicating that they had different adaptations to UVB irradiation. The adaptation of males to radiation compared with females is important in shortening the duration of the development of *T. drosophilae* and guiding the separation of males and females during mass rearing.

As an important indicator of parasitoids for biological control, obtaining as many females as possible can improve the efficiency of pest control, and it is also an important guide for the mass rearing of parasitoids [70,71,72,73]. The sex ratio of UVB-irradiated hosts supplied with *T. drosophilae* increased with radiation time to a maximum of 7 h. Changing temperatures are also a key factor for the effectiveness of control when carrying out field applications of parasitoids. At the same time, temperature is also an important factor in the sex ratio [74]. A temperature of 26 °C is the highest reproductive temperature for the offspring sex ratio of *Spalangia endius* Walker (Hymenoptera: Pteromalidae) [75]. The sex ratio of *Diversinervus elegans* Silvestri (Hymenoptera: Encyrtidae) decreased gradually with the increase in temperature in the range of 18–30 °C, and the female ratio of its progeny reached 74.24% under 18 °C [76]. In our study, UVB radiation was observed to affect the sex ratio of parasitoids and is thus a potential method for female acquisition. Taken together, the increase in the sex ratio due to radiation seems to have been at the expense of male death or non-emergence [77]. This combination of low temperature and UVB radiation leading to parasitoid feminization is significant for guiding irradiation of pupae in mass rearing parasitoids for field applications, and it is worth further investigating the treatments selected in this study in combination with low-temperature refrigeration techniques [40,41,51,78]. The mechanisms controlling the sex ratio of parasitoids are extremely complex, and they include adult parasitoid age, mating status, nutrient supply, genetics, host, and environment, which all affect the sex ratio of parasitoids [79,80,81,82]. This study only investigated the effects of ultraviolet radiation and outdoor environmental conditions on the sex ratio of parasitoids, while the interaction between other factors and underlying mechanisms needs to be further explored [83,84,85,86,87,88]. This study reveals their comprehensive influence on the sex ratio and its internal mechanism.

The results of the outdoor quality tests of F1 and F2 generations of *T. drosophilae* show that approximately the same quality of parasitoids emerged from irradiated and normal pupae. The index values were higher for most treatments than for the control, indicating that irradiated hosts are able to successfully search for hosts and complete normal parasitization before emerging under outdoor test conditions and can thus be directly applied in field prevention and control. However, there are also a lot of criticisms, and it will be very important to address these in future work. As a rather important aspect, irradiation also has certain drawbacks; for example, the pupae of flies have a high death rate after radiation. In the process of mass rearing parasitoids, a high rate of dead pupae will increase reproduction cost [89,90,91]. At the same time, the collected pupae will be wasted, and reproduction efficiency will be reduced [92]. If we can streamline the collection of pupae from *D. suzukii*, we can then consider using it as a host to try to minimize these losses. At the same time, the host range available for large-scale rearing of parasitoids will be expanded, which maximizes the effectiveness of biological control and minimizes the cost. Therefore, all aspects need to be taken into account when evaluating and selecting radiation conditions. It is expected that the process can be streamlined while improving reproductive efficiency and economic benefits. We expect to realize the vision of providing more high-quality and efficient parasitoids with great outdoor adaptability for the biological control of *Drosophila.*

## 5. Conclusions

Based on the results of this study, and as described above, ultraviolet radiation was shown to affect the parasitic efficacy and outdoor performance of *T. drosophilae*. The research results provide a theoretical basis for further clarification of the application of ultraviolet irradiation in the field of pest control. Testing the outdoor performance of parasitoids can unlock the full potential of resources for the biological control of pest. It also can optimize long-lasting pest control strategies in the field to protect the environment and achieve sustainable development.

## Figures and Tables

**Table 1:** Quality of *Trichopria drosophilae* (F1 and F2) that emerged from host pupae irradiated at 6 h in outside test based on parasitism rate, emergence rate, sex ratio, and pupal death rate.

Indexes	Treatments and Generations		
	CK	F1	F2
Parasitism rate (%)	48.00 ± 0.01 b	59.67 ± 0.02 a	51.67 ± 0.01 b
Emergence rate (%)	46.67 ± 0.00 b	59.67 ± 0.02 a	51.67 ± 0.01 a
Sex ratio (♀/♂)	1.84 ± 0.18 b	3.20 ± 0.25 a	2.46 ± 0.18 b
Pupal death rate (%)	13.33 ± 0.01 b	29.67 ± 0.02 a	13.33 ± 0.00 b

Note: In this table and the subsequent table, data are presented as mean ± SD; data with different lowercase letters indicated a significant difference at 0.05 level. Here and below, CK means control with no irradiated treatment.

**Table 2:** Quality of *Trichopria drosophilae* (F1 and F2) that emerged from host pupae irradiated at 6 h in outside test based on development duration (DD) and longevity (L).

Indexes (Days)	Treatments and Generations			
	F1		F2	
	CK	Ultraviolet-B	CK	Ultraviolet-B
Female DD	21.30 ± 0.67 b	23.80 ± 0.96 a	21.30 ± 0.67 b	20.70 ± 0.88 b
Male DD	17.30 ± 0.83 a	16.10 ± 0.78 a	17.30 ± 0.83 a	17.50 ± 0.93 a
Female L	52.10 ± 1.55 a	54.93 ± 1.33 a	52.10 ± 1.55 a	53.07 ± 1.67 a
Male L	35.87 ± 1.84 a	40.87 ± 1.75 a	35.87 ± 1.84 a	37.93 ± 1.91 a

## Data Availability

The datasets in this study are available from the corresponding author on reasonable request.

## References

[1] Schneider D. (2000). Using *Drosophila* as a model insect. Nat. Rev. Genet..

[2] Sharma V., Mishra A.K., Mutsuddi M., Mukherjee A., Mutsuddi M., Mukherjee A. (2019). Mighty fly: An introduction to *Drosophila*. Insights into Human Neurodegeneration: Lessons Learnt from Drosophila.

[3] Garcia F.R.M., Garcia F.R.M. (2021). Introduction to *Drosophila suzukii* management. Drosophila suzukii Management.

[4] Hamby K.A., Bellamy D., Chiu J.C., Lee J.C., Walton V.M., Wiman N.G., York R.M., Biondi A. (2016). Biotic and abiotic factors impacting development, behavior, phenology, and reproductive biology of *Drosophila suzukii*. J. Pest Sci..

[5] Walsh D.B., Bolda M.P., Goodhue R.E., Dreves A.J., Lee J., Bruck D.J., Walton V.M., O’Neal S.D., Zalom F.G. (2011). *Drosophila suzukii* (Diptera: Drosophilidae): Invasive pest of ripening soft fruit expanding its geographic range and damage potential. J. Integr. Pest Manag..

[6] Goodhue R.E., Bolda M., Farnsworth D., Williams J.C., Zalom F.G. (2011). Spotted wing drosophila infestation of California strawberries and raspberries: Economic analysis of potential revenue losses and control costs. Pest Manag. Sci..

[7] Garcia F.R.M., Lasa R., Funes C.F., Buzzetti K. (2022). *Drosophila suzukii* management in Latin America: Current status and perspectives. J. Econ. Entomol..

[8] Tochen S., Dalton D.T., Wiman N., Hamm C., Shearer P.W., Walton V.M. (2014). Temperature-related development and population parameters for *Drosophila suzukii* (Diptera: Drosophilidae) on cherry and blueberry. Environ. Entomol..

[9] Wiman N.G., Walton V.M., Dalton D.T., Anfora G., Burrack H.J., Chiu J.C., Daane K.M., Grassi A., Miller B., Tochen S. (2014). Integrating temperature-dependent life table data into a matrix projection model for *Drosophila suzukii* population estimation. PLoS ONE.

[10] Dam D., Molitor D., Beyer M. (2019). Natural compounds for controlling *Drosophila suzukii*. A review. Agron. Sustain. Dev..

[11] Shawer R., Garcia F.R.M. (2020). Chemical control of *Drosophila suzukii*. Drosophila suzukii Management.

[12] Shawer R., Tonina L., Tirello P., Duso C., Mori N. (2018). Laboratory and field trials to identify effective chemical control strategies for integrated management of *Drosophila suzukii* in European cherry orchards. Crop Prot..

[13] Kruitwagen A., Beukeboom L.W., Wertheim B. (2018). Optimization of native biocontrol agents, with parasitoids of the invasive pest *Drosophila suzukii* as an example. Evol. Appl..

[14] Gabarra R., Riudavets J., Rodríguez G.A., Pujade-Villar J., Arnó J. (2015). Prospects for the biological control of *Drosophila suzukii*. BioControl.

[15] Miller B., Anfora G., Buffington M., Daane K.M., Dalton D.T., Hoelmer K.M., Rossi Stacconi M.V., Grassi A., Ioriatti C., Loni A. (2015). Seasonal occurrence of resident parasitoids associated with *Drosophila suzukii* in two small fruit production regions of Italy and the USA. Bull. Insectol..

[16] Cancino M.D.G., Hernández A.G., Cabrera J.G., Carrillo G.M., González J.A.S., Bernal H.C.A. (2015). Parasitoides de *Drosophila suzukii* (Matsumura) (Diptera: Drosophilidae) en Colima, México. Southwest. Entomol..

[17] Wang X.G., Kaçar G., Biondi A., Daane K.M. (2016). Life-history and host preference of *Trichopria drosophilae*, a pupal parasitoid of spotted wing drosophila. BioControl.

[18] Yi C.D., Cai P.M., Lin J., Liu X.X., Ao G.F., Zhang Q.W., Xia H.M., Yang J.Q., Ji Q.E. (2020). Life history and host preference of *Trichopria drosophilae* from southern China, one of the effective pupal parasitoids on the *Drosophila* species. Insects.

[19] Wang X.G., Kaçar G., Biondi A., Daane K.M. (2016). Foraging efficiency and outcomes of interactions of two indigenous parasitoids attacking the invasive spotted wing drosophila. Biol. Control.

[20] Liu B., Li Y.Y., Xiong Y., Liu S.N., Hu C.H., Xiao C., Tang G.W. (2017). Mating behavior of *Trichopria drosophilae* and the effect of male mating frequency on the production of female offspring. Chin. J. Appl. Entomol..

[21] Wang X.G., Nance A.H., Jones J.M.L., Hoelmer K.A., Daane K.M. (2018). Aspects of the biology and reproductive strategy of two Asian larval parasitoids evaluated for classical biological control of *Drosophila suzukii*. Biol. Control.

[22] Wang X.G., Serrato M.A., Son Y., Walton V.M., Hogg B.N., Daane K.M. (2018). Thermal performance of two indigenous pupal parasitoids attacking the invasive *Drosophila suzukii* (Diptera: Drosophilidae). Environ. Entomol..

[23] Chabert S., Allemand R., Poyet M., Eslin P., Gibert P. (2012). Ability of European parasitoids (Hymenoptera) to control a new invasive Asiatic pest, *Drosophila suzukii*. Biol. Control.

[24] Kremmer L., Thaon M., Borowiec N., David J., Poirié M., Gatti J.L., Ris N. (2017). Field monitoring of *Drosophila suzukii* and associated communities in south eastern France as a pre-requisite for classical biological control. Insects.

[25] Daane K.M., Wang X.G., Biondi A., Miller B., Miller J.C., Riedl H., Shearer P.W., Guerrieri E., Giorgini M., Buffington M. (2016). First exploration of parasitoids of *Drosophila suzukii* in South Korea as potential classical biological agents. J. Pest Sci..

[26] Boycheva W.S., Romeis J., Collatz J. (2019). Influence of the rearing host on biological parameters of *Trichopria drosophilae*, a potential biological control agent of *Drosophila suzukii*. Insects.

[27] Dai X.Y., Shi H.M., Jiang X.L., Liang H.M., Guo Y.Y., Gao H.H. (2019). Host stage preference of *Trichopria drosophilae* to fruit fly pupae. Chin. J. Biol. Control.

[28] Chen J.N., Zhou S.C., Wang Y., Shi M., Chen X.X., Huang J.H. (2018). Biocontrol characteristics of the fruit fly pupal parasitoid *Trichopria drosophilae* (Hymenoptera: Diapriidae) emerging from different hosts. Sci. Rep..

[29] Rossi S.M.V., Grassi A., Ioriatti C., Anfora G. (2019). Augmentative releases of *Trichopria drosophilae* for the suppression of early season *Drosophila suzukii* populations. BioControl.

[30] Zhu C.J., Li J., Wang H., Zhang M., Hu H.Y. (2017). Demographic potential of the pupal parasitoid *Trichopria drosophilae* (Hymenoptera: Diapriidae) reared on *Drosophila suzukii* (Diptera: Drosophilidae). J. Asia-Pac. Entomol..

[31] Zhang J., Wang F., Yuan B., Yang L., Yang Y., Fang Q., Kuhn J.H., Song Q.S., Ye G.Y. (2021). A novel cripavirus of an ectoparasitoid wasp increases pupal duration and fecundity of the wasp’s *Drosophila melanogaster* host. ISME J..

[32] Huang J.H., Chen J.N., Fang G.Q., Pang L., Zhou S.C., Zhou Y.N., Pan Z.Q., Zhang Q.C., Sheng Y.F., Lu Y.Q. (2021). Two novel venom proteins underlie divergent parasitic strategies between a generalist and a specialist parasite. Nat. Commun..

[33] Chen J.N., Fang G.Q., Pang L., Sheng Y.F., Zhang Q.C., Zhou Y.N., Zhou S.C., Lu Y.Q., Liu Z.G., Zhang Y.X. (2021). Neofunctionalization of an ancient domain allows parasites to avoid intraspecific competition by manipulating host behaviour. Nat. Commun..

[34] Herz A., Dingeldey E., Englert C. (2021). More power with flower for the pupal parasitoid *Trichopria drosophilae*: A candidate for biological control of the Spotted Wing Drosophila. Insects.

[35] Liu X.X., Yi C.D., Ao G.F., Chen S., Ji Q.E. (2021). Effects of three kinds of ultraviolet radiation on pupae of *Drosophila melanogaster*. Chin. J. Biol. Control.

[36] Van Atta K.J., Potter K.A., Woods H.A. (2015). Effects of UV-B on environmental preference and egg parasitization by *Trichogramma* wasps (Hymenoptera: Trichogrammatidae). J. Entomol. Sci..

[37] Alwandri H., Kusumawati N., Sudaryadi I. (2022). The Effect of UV Radiation and Fruit Feedings (Banana and Guava) on the Survival Rate and Morphological Changes of Reproductive Organ of Fruit Fly (*Drosophila melanogaster* Meigen, 1830). Proceedings of the 7th International Conference on Biological Science (ICBS 2021).

[38] Ayvaz A., Karasu E., Karabörklü S., Tunçbilek A.Ş. (2008). Effects of cold storage, rearing temperature, parasitoid age and irradiation on the performance of *Trichogramma evanescens* Westwood (Hymenoptera: Trichogrammatidae). J. Stored Prod. Res..

[39] Masry S.H., El-Wakeil N., El-Wakeil N., Saleh M., Abu-hashim M. (2020). Egg parasitoid production and their role in controlling insect pests. Cottage Industry of Biocontrol Agents and Their Applications.

[40] Shinde C.U., Radadia G.G., Ghetiya L.V., Shah K.D., Gadhiya V.C., Patel A.D. (2016). Effect of non-ionizing (UV) radiation on the development of egg parasitoid, *Trichogramma chilonis* Ishii (Hymenoptera: Trichogrammatidae). Adv. Life Sci..

[41] St-Onge M., Cormier D., Todorova S., Lucas E. (2016). Conservation of *Ephestia kuehniella* eggs as hosts for *Trichogramma ostriniae*. J. Appl. Entomol..

[42] St-Onge M., Cormier D., Todorova S., Lucas É. (2014). Comparison of *Ephestia kuehniella* eggs sterilization methods for *Trichogramma* rearing. Biol. Control.

[43] Xu J., Yang X., Lin Y., Zang L.S., Tian C.Y., Ruan C.C. (2016). Effect of fertilized, unfertilized, and UV-irradiated hosts on parasitism and suitability for *Trichogramma* parasitoids. Entomol. Exp. Appl..

[44] Mawela K.V., Kfir R., Krüger K. (2010). Host suitability of UV-irradiated eggs of three Lepidoptera species for rearing *Trichogrammatoidea lutea* Girault (Hymenoptera: Trichogrammatidae). J. Appl. Entomol..

[45] Foggo A., Higgins S., Wargent J.J., Coleman R.A. (2007). Tri-trophic consequences of UV-B exposure: Plants, herbivores and parasitoids. Oecologia.

[46] Dhal M.K., Seth R.K. (2017). Effect of ionizing (gamma) and non-ionizing (UV) radiation on Lepidopteran host eggs for the efficacy of egg parasitoid, *Trichogramma Chilonis* Ishii (Hymenoptera: Trichogrammatidae). Int. J. Res. Biosci. Agric. Technol..

[47] Little C.M., Rizzato A.R., Charbonneau L., Chapman T., Hillier N.K. (2019). Color preference of the spotted wing Drosophila, *Drosophila suzukii*. Sci. Rep..

[48] Renkema J.M., Iglesias L.E., Bonneau P., Liburd O.E. (2018). Trapping system comparisons for and factors affecting populations of *Drosophila suzukii* and *Zaprionus indianus* in winter-grown strawberry. Pest Manag. Sci..

[49] Renkema J.M., Buitenhuis R., Hallett R.H. (2014). Optimizing trap design and trapping protocols for *Drosophila suzukii* (Diptera: Drosophilidae). J. Econ. Entomol..

[50] Iglesias L.E., Nyoike T.W., Liburd O.E. (2014). Effect of trap design, bait type, and age on captures of *Drosophila suzukii* (Diptera: Drosophilidae) in berry crops. J. Econ. Entomol..

[51] Zapater M.C., Andiarena C.E., Camargo G.P., Bartoloni N. (2009). Use of irradiated *Musca domestica* pupae to optimize mass rearing and commercial shipment of the parasitoid *Spalangia endius* (Hymenoptera: Pteromalidae). Biocontrol Sci. Technol..

[52] Peng W., Li Y.X., Weng S.H., Zhou R.H., Pan X., Li J.Y., Han B.Y. (2020). Progress and prospects of sex-separation techniques for dipteran insects. Acta Entomol. Sin..

[53] Zhang X., Yi P., Chu P.F., Yuan X.Q., Zhan E.L., Leng C.M., Li Y., Hu D., Li Y.P. (2020). Effects of microwave irradiation on the growth, development andreproduction of the green peach aphid, *Myzus persicae* (Hemiptera: Aphididae). Acta Entomol. Sin..

[54] Ben-Yakir D., Fereres A. (2016). The effects of UV radiation on arthropods: A review of recent publications (2010–2015). International Symposium on Light in Horticulture.

[55] Tilton E.W., Brower J.H. (2018). Radiation effects on arthropods. Preservation of Food by Ionizing Radiation.

[56] Tuncbilek A.S., Kusmus A., Canpolat U.A., Ercan F.S. (2011). Effect of UV radiation on *Bracon Hebetor* (Hymenoptera: Braconidae) and its host larvae, *Ephestia Kuehniella* (Lepidoptera: Pyralidae). Ann. Univ. Craiova-Agric. Mont. Cadastre Ser..

[57] Cui H.Y., Zeng Y.Y., Reddy G.V., Gao F., Li Z.H., Zhao Z.H. (2021). UV radiation increases mortality and decreases the antioxidant activity in a tephritid fly. Food Energy Secur..

[58] Espo E., Eyidozehi K., Ravan S. (2015). Influence of gamma and ultraviolet irradiation on pest control. MAGNT Res. Rep..

[59] Shimoda M., Honda K.I. (2013). Insect reactions to light and its applications to pest management. Appl. Entomol. Zool..

[60] Nakagawa S., Farias G.J., Steiner L.F. (1970). Response of female Mediterranean fruit flies to male lures in the relative absence of males. J. Econ. Entomol..

[61] Jarrett B.J., Linder S., Fanning P.D., Isaacs R., Szűcs M. (2022). Experimental adaptation of native parasitoids to the invasive insect pest, *Drosophila suzukii*. Biol. Control.

[62] Lee J.C., Wang X.G., Daane K.M., Hoelmer K.A., Isaacs R., Sial A.A., Walton V.M. (2019). Biological control of spotted-wing Drosophila (Diptera: Drosophilidae)-current and pending tactics. J. Integr. Pest Manag..

[63] Wolf S., Boycheva-Woltering S., Romeis J., Collatz J. (2020). *Trichopria drosophilae* parasitizes *Drosophila suzukii* in seven common non-crop fruits. J. Pest Sci..

[64] Häussling B.J., Mautner M., Stökl J. (2022). Below ground efficiency of a parasitic wasp for *Drosophila suzukii* biocontrol in different soil types. Sci. Rep..

[65] Wang L., Zhu F., Lei Z.L. (2015). Effects of UV radiation on biological characteristics of *Plutella xylostella*. Stud. Insects Cent. China.

[66] Carton Y., Poirié M., Nappi A.J. (2008). Insect immune resistance to parasitoids. Insect Sci..

[67] Douglas A.E. (2015). Multiorganismal insects: Diversity and function of resident microorganisms. Annu. Rev. Entomol..

[68] Mateos M., Winter L., Winter C., Higareda-Alvear V.M., Martinez-Romero E., Xie J. (2016). Independent origins of resistance or susceptibility of parasitic wasps to a defensive symbiont. Ecol. Evol..

[69] Ventura I.M., Martins A.B., Lyra M.L., Andrade C.A., Carvalho K.A., Klaczko L.B. (2012). Spiroplasma in *Drosophila melanogaster* populations: Prevalence, male-killing, molecular identification, and no association with *Wolbachia*. Microb. Ecol..

[70] Van G.J., Herre E.A., Gómez A., Nason J.D. (2022). Extraordinarily precise nematode sex ratios: Adaptive responses to vanishingly rare mating opportunities. Proc. R. Soc. B.

[71] Krüger A.P., Scheunemann T., Vieira J.G.A., Morais M.C., Bernardi D., Nava D.E., Garcia F.R.M. (2019). Effects of extrinsic, intraspecific competition and host deprivation on the biology of *Trichopria anastrephae* (Hymenoptera: Diapriidae) reared on *Drosophila suzukii* (Diptera: Drosophilidae). Neotrop. Entomol..

[72] Linder S., Jarrett B.J., Fanning P., Isaacs R., Szűcs M. (2022). Limited gains in native parasitoid performance on an invasive host beyond three generations of selection. Evol. Appl..

[73] Wolf S., Barmettler E., Eisenring M., Romeis J., Collatz J. (2021). Host searching and host preference of resident pupal parasitoids of *Drosophila suzukii* in the invaded regions. Pest Manag. Sci..

[74] Stacconi M.V.R., Panel A., Baser N., Ioriatti C., Pantezzi T., Anfora G. (2017). Comparative life history traits of indigenous Italian parasitoids of *Drosophila suzukii* and their effectiveness at different temperatures. Biol. Control.

[75] Liu X. (2017). Study on Reproductive Biology of *Spalangia endius* Walker. Master’s Thesis.

[76] Wang J.Q., Xu L.Y., Li F.C., Zheng Y.P., Deng Y.X., Zhang Y.K., Zhu G.Y., Li G.H. (2019). Effect of temperature on emergence rate and sex ratio of *Diversinervus elegans* Silvestri. J. Environ. Entomol..

[77] Hallouti A., Ait H.M., Tazi H., Ait H.R., Zahidi A., Ait B.A.A., Boubaker H. (2021). Pathogenicity of *Fusarium* spp. isolates against the Mediterranean fruit fly (*Ceratitis capitata*) and their responses to ultraviolet-B radiation and water stress. Entomol. Exp. Appl..

[78] Ghosh E., Ballal C.R. (2018). Short-term storage of the egg parasitoids, *Trichogramma* and *Trichogrammatoidea*. Egypt. J. Biol. Pest Control.

[79] László Z., Dénes A.L., Király L., Tóthmérész B. (2018). Biased parasitoid sex ratios: *Wolbachia*, functional traits, local and landscape effects. Basic Appl. Ecol..

[80] Tang Y.L., Wang L.N., Wang Y.Q., Zhang Y.L., Wang X.Y., Wei K. (2022). Effects of different foundress densities on sex ratio of the offspring of Bethylid wasps. Sci. Silvae Sin..

[81] Huang J., Zhi F.Y., Lv Y.B. (2019). Fitness of *Aenasius bambawalei* (Hymenoptera: Encyrtidae) on a new host species. Acta Entomol. Sin..

[82] Hu S., Luo L.P., Wang X.Y. (2019). Wing dimorphism and sex ratio changes in progeny of various sister broods in parasitoid *Sclerodermus pupariae* (Hymenoptera: Bethylidae). For. Res..

[83] Krylova A.E., Chaplygina A.V., Vekshin N.L. (2020). Formation of Lipofuscin in *Drosophila* after exposure to elevated temperatures and UV radiation. Biophysics.

[84] Bath E., Edmunds D., Norman J., Atkins C., Harper L., Rostant W.G., Chapman T., Wigby S., Perry J.C. (2021). Sex ratio and the evolution of aggression in fruit flies. Proc. R. Soc. B.

[85] Alvarenga C.D., Dias V., Stuhl C., Sivinski J. (2016). Contrasting brood-sex ratio flexibility in two Opiine (Hymenoptera: Braconidae) parasitoids of tephritid (Diptera) fruit flies. J. Insect Behav..

[86] West S.A., Reece S.E., Sheldon B.C. (2002). Sex ratios. Heredity.

[87] Werren J.H. (1980). Sex ratio adaptations to local mate competition in a parasitic wasp. Science.

[88] Ghana S., Suleman N., Compton S.G. (2012). Factors influencing realized sex ratios in fig wasps: Double oviposition and larval mortalities. J. Insect Behav..

[89] Rempoulakis P., Castro R., Nemny-Lavy E., Nestel D. (2015). Effects of radiation on the fertility of the Ethiopian fruit fly, *Dacus ciliatus*. Entomol. Exp. Appl..

[90] Paithankar J.G., Deeksha K., Patil R.K. (2017). Gamma radiation tolerance in different life stages of the fruit fly *Drosophila melanogaster*. Int. J. Radiat. Biol..

[91] Collins S.R., Weldon C.W., Banos C., Taylor P.W. (2008). Effects of irradiation dose rate on quality and sterility of Queensland fruit flies, *Bactrocera tryoni* (Froggatt). J. Appl. Entomol..

[92] Mastrangelo T., da Silva F.F., Mascarin G.M., da Silva C.B. (2019). Multispectral imaging for quality control of laboratory-reared *Anastrepha fraterculus* (Diptera: Tephritidae) pupae. J. Appl. Entomol..

